# Lynch Syndrome-Related Clear Cell Carcinoma of the Cervix: A Case Report

**DOI:** 10.3390/ijms19040979

**Published:** 2018-03-25

**Authors:** Kohei Nakamura, Kentaro Nakayama, Toshiko Minamoto, Tomoka Ishibashi, Kaori Ohnishi, Hitomi Yamashita, Ruriko Ono, Hiroki Sasamori, Sultana Razia, Mohammad Mahmud Hossain, Shanta Kamrunnahar, Masako Ishikawa, Noriyoshi Ishikawa, Satoru Kyo

**Affiliations:** 1 Department of Obstetrics and Gynecology, Shimane University School of Medicine, Enyacho 89-1, Izumo 6938501, Japan; kohei320@med.shimane-u.ac.jp (K.N.); tokominmin@gmail.com (T.M.); tomoka19850314@gmail.com (T.I.); kaorisanuki@gmail.com (K.O.); memedasudasu1103@gmail.com (H.Y.); ruriko.u.u.0815@gmail.com (R.O.); sasaringo22@yahoo.co.jp (H.S.); raeedahmed@yahoo.com (S.R.); likhon.vet@gmail.com (M.M.H.); shanta.vet@gmail.com (S.K.); pcbashi4@yahoo.co.jp (M.I.); satoruky@med.shimane-u.ac.jp (S.K.); 2 Department of Organ Pathology, Shimane University School of Medicine, Izumo 6938501, Japan; kanatomo@med.shimane-u.ac.jp

**Keywords:** Lynch syndrome, cervical cancer, immunohistochemistry

## Abstract

Lynch syndrome, a hereditary cancer syndrome, occurs because of germline mutations in at least one of four DNA mismatch repair genes (MutL Homolog 1 (*MLH1*), MutS Homolog 2 (*MSH2*), MutS Homolog 6 (*MSH6*), and PMS1 Homolog 2 (*PMS2*)). The disorder is associated with colorectal, endometrial, and other epithelial malignancies, but not cervical cancer. We report a woman with Lynch syndrome with synchronous cervical cancer. This is the first report of Lynch syndrome-related clear cell carcinoma of the cervix, which indicates the possibility of an association between cervical cancer and Lynch syndrome. Suitable genetic tests are required to determine whether common genetics can account for synchronous or subsequent malignancies in Lynch syndrome patients and their families. Such knowledge will also enhance our understanding of the genetic mechanisms governing the development of apparently unrelated cancers.

## 1. Introduction

Lynch syndrome (also called hereditary non-polyposis colorectal cancer) is an autosomal dominant disorder that arises because of a germline mutation in at least one of four DNA mismatch repair (MMR) genes (MutL Homolog 1 (*MLH1*), MutS Homolog 2 (*MSH2*), MutS Homolog 6 (*MSH6*), and PMS1 Homolog 2 (*PMS2*)). Lynch syndrome is associated with the microsatellite instability (MSI)-high phenotype [1]. Individuals who carry MMR mutations are at higher risk of developing a number of epithelial malignancies, such as colorectal, endometrial, ovarian, stomach, pancreas, small intestine, renal, ureteral, and brain cancer [2,3,4,5]. MMR gene mutations can be detected by a loss of expression of MMR proteins by immunohistochemistry. There is some evidence that MMR gene mutation carriers might be at increased risk of cervical cancer; however, it is currently uncertain whether cervical cancer is associated with Lynch syndrome. Herein, we report a case of Lynch syndrome-related clear cell carcinoma of the cervix with synchronous colorectal cancer, which could be distinguished from non-Lynch syndrome-related cervical cancer by immunohistochemical detection of MMR genes and MSI analysis.

## 2. Case

The study protocol was approved by the Ethics Committee of Shimane University Faculty of Medicine, Izumo, Japan (no. 960). The patient provided informed, written consent. A 42-year-old gravida 2, para 2 woman presented with abnormal vaginal bleeding in April 2017. Her medical history and family history were unremarkable. Levels of serum carbohydrate antigen 125 (CA 125), CA 19-9, carcinoembryonic antigen, and squamous cell carcinoma antigen were within the normal range. Her general examination was similarly unremarkable.

**Figure 1:**
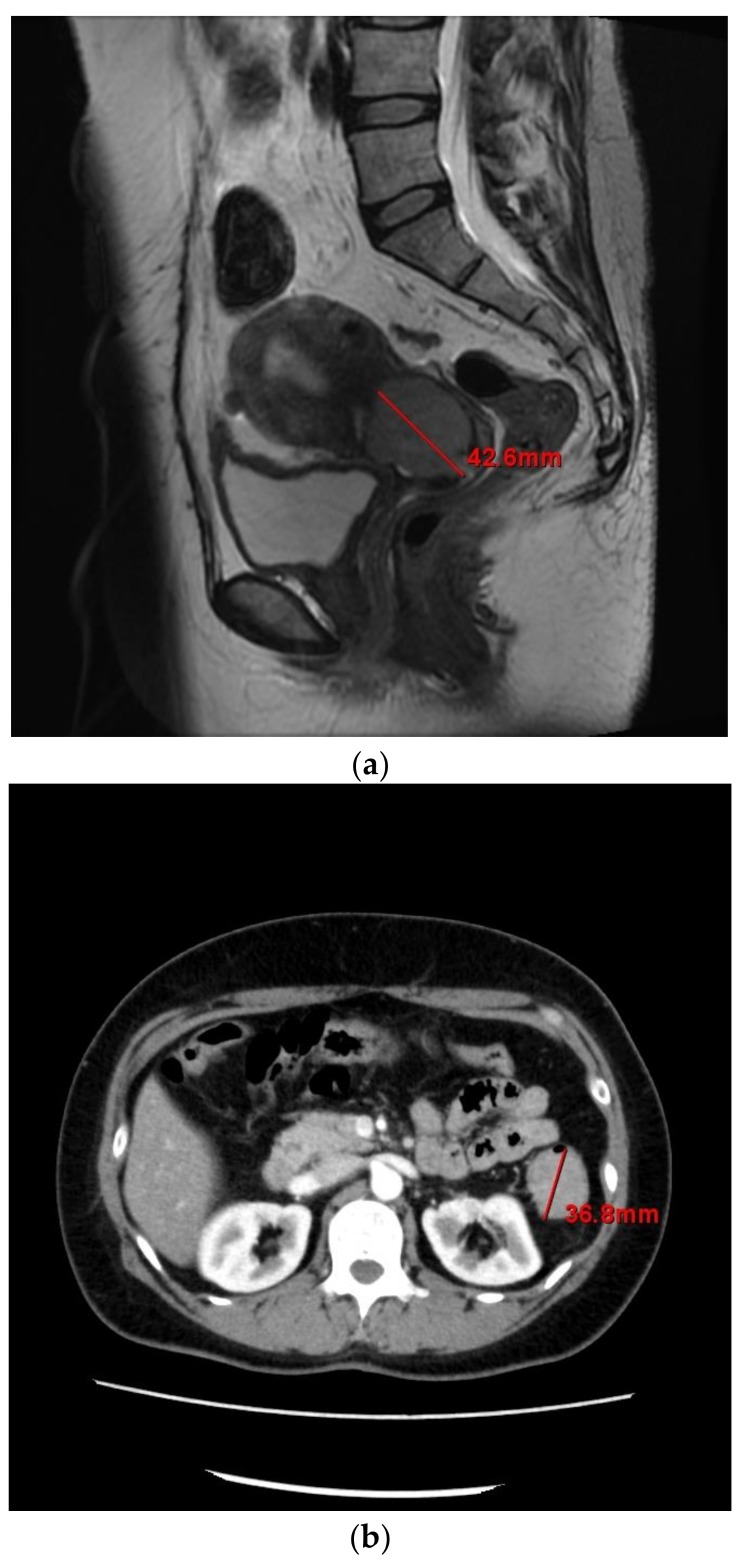
(**a**) Pelvic magnetic resonance imaging of the tumor in the endocervix; (**b**) Contrast-enhanced computed tomography of the abdomen showing the transverse colon tumor.

Pelvic magnetic resonance imaging indicated a tumor in the endocervix measuring 42 × 32 × 38 mm (Figure 1a), and a tumor in the transverse colon measuring 36 mm in the largest diameter (Figure 1b). It did not show peritonitis carcinomatosa, ascites, dissemination nodules, or metastasis to other organs. Contrast-enhanced computed tomography (CT) showed no lymphadenopathy. These findings suggested colorectal metastasis from cervical cancer or double cancer (cervical and colorectal cancer). Positron emission tomography-CT showed abnormal ^18^F-fluorodeoxyglucose uptake in the cervix and colorectum.

Endoscopic submucosal dissection was performed for the colorectal tumor in June 2017. The resected specimen was diagnosed as well to moderately differentiated adenocarcinoma (pT1N0M0) with negative margins. Next, radical hysterectomy, bilateral salpingo-oophorectomy, pelvic lymphadenectomy, and left hemicolectomy were performed in July 2017. After surgery, there was no visible residual disease in the pelvic region. The resected specimens in the cervix were reviewed by a pathologist and the mass was finally diagnosed as a 45 × 40 × 15-mm clear cell carcinoma of uterine cervix with lymphovascular invasion (pT2bN0M0). No tumor remained in the resected specimen in the colorectum. These findings confirmed double cancer.

The patient experienced an uneventful postoperative period and was discharged from the hospital on the 14th day after surgery. She was administered combination chemotherapy (paclitaxel 175 mg/m^2^ + carboplatin 700 mg/body) after the surgery and is tolerating her chemotherapy well and has remained asymptomatic and progression-free for eight months.

### 2.1. Pathological Findings

**Figure 2:**
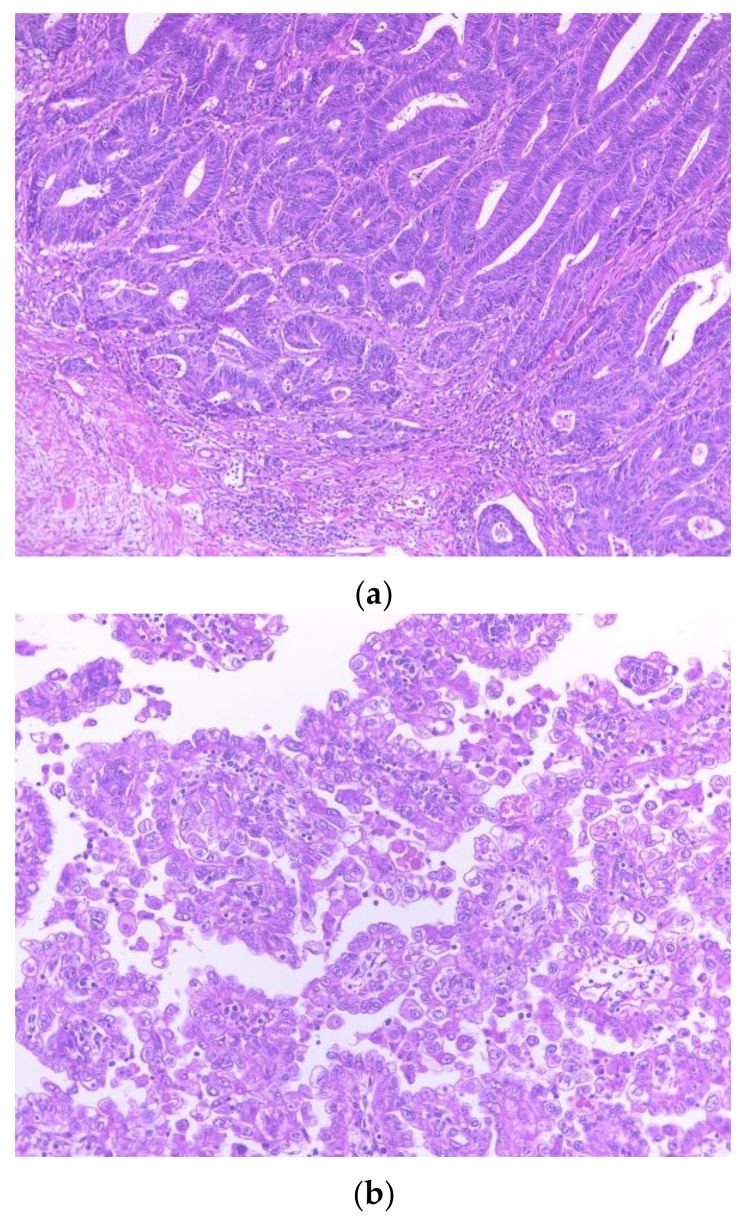
(**a**) Colon carcinoma showing invasion into the submucosa (hematoxylin and eosin, 200×); (**b**) Hematoxylin and eosin staining of the cervical clear cell carcinoma. The glycogen-rich clear cells cytoplasm with high-grade nuclear atypia (400×).

The colonic specimen showed that the tumor had invaded the submucosa, and lymphatic invasion was also identified (Figure 2a). The cervical tumor showed a tubular pattern with a lining composed of hobnail, flat, and clear cells. There was also a papillotubular pattern visible with focal solid areas with a lining of glycogen-rich clear cell cytoplasm, as well as high-grade nuclear atypia (Figure 2b). Histological examination revealed the absence of malignant cells in all resected samples, including the endometrium, ovaries, oviduct, cervical stump, parametria, vaginal cuff, infundibular pelvic ligaments, and pelvic nodes.

### 2.2. Immunohistochemical and MSI Analysis Findings

The colonic tumor was positive for MLH1 and PMS2, and showed no expression of MSH2 or MSH6, which was in agreement with the germline mutations in *MSH2* and *MSH6*.

In the cervical tumor, the epithelial-like cells were strongly positive for hepatocyte nuclear factor 1β and p16. Napsin A was weakly expressed. The cells were negative for estrogen receptor, progesterone receptor, and vimentin. With regard to MMR gene expression, the tumor was positive for MLH1 and PMS2, and showed no expression of MSH2 or MSH6, which was in agreement with the germline mutation in *MSH2* and *MSH6* and consistent with the results from the colonic tumor.

**Figure 3:**
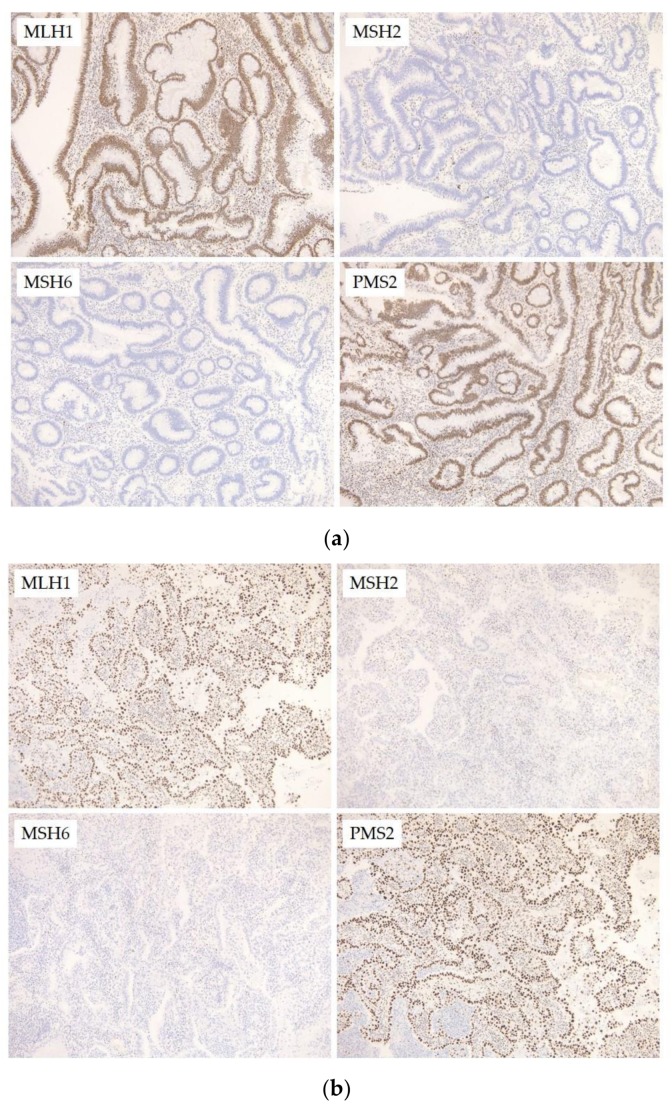

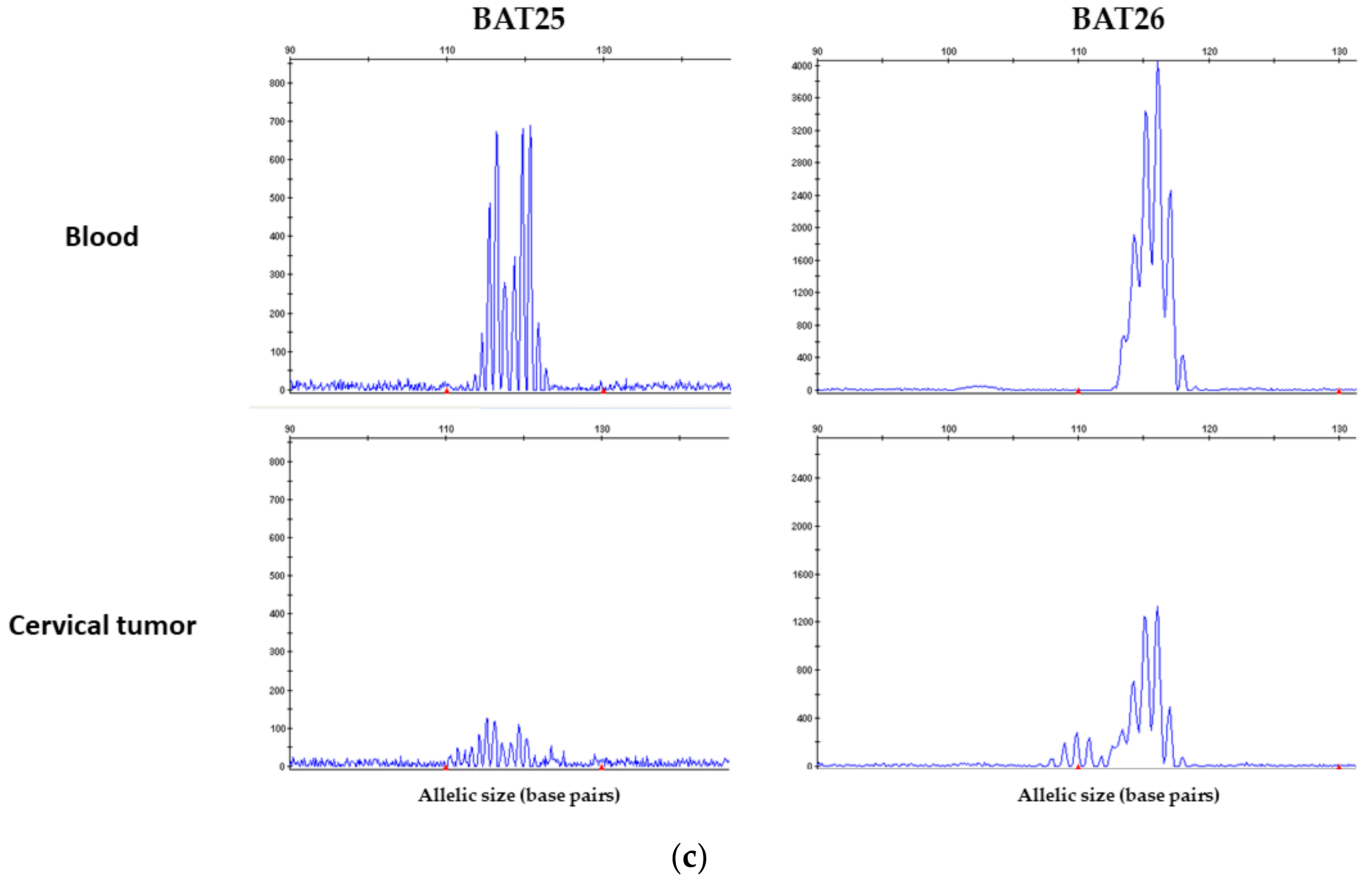
(**a**) Immunohistochemical staining of the colon carcinoma. The colon carcinoma was negative for MSH2 (MutS Homolog 2) and MSH6 (MutS Homolog 6) and positive for MLH1 (MutL Homolog 1) and PMS2 (PMS1 Homolog 2) (200×), which was in agreement with the germline mutation in *MSH2* and *MSH6*; (**b**) Immunohistochemical staining of the cervical carcinoma. The cervical carcinoma was negative for MSH2 and MSH6 and positive for MLH1 and PMS2 (200×), which was in agreement with the germline mutation in *MSH2* and *MSH6*; (**c**) Microsatellite instability analysis of the cervical carcinoma. Allele length patterns of eight mononucleotide markers in control (**top**) and cervical tumor (**bottom**) tissues; two microsatellite markers (BAT25 and BAT26) show instability, visible as the shift in the size (base pairs) of the amplification products in the cervical tumor sample (BAT25) and new alleles in the cervical tumor sample (BAT26).

For MSI analysis, genomic DNA was extracted from fresh tissue from the cervical tumor and blood samples using the QIAamp DNA Micro Kit (Qiagen, Valencia, CA, USA) according to the manufacturer’s standard protocol. We performed MSI analysis using the polymerase chain reaction (PCR) with eight microsatellite markers (BAT25, BAT26, D2S123, D5S346, D17S250, NR21, MONO27, and NR2). MSI analysis in the cervical tumor and blood using the National Cancer Institute panel revealed that the tumor had high MSI. Particularly, the cervical tumor showed instability in two microsatellite repeats (Figure 3c), which is considered MSI-high.

## 3. Discussion

This study presents a fascinating case of Lynch syndrome-related colon cancer with synchronous clear cell carcinoma of the cervix.

Few case reports have described Lynch-related endocervical adenocarcinoma, the typical cervical cancer observed in women believed or shown to have MMR gene mutations [6,7,8]. One case reported a germline mutation in *MSH2*, in which the tumor was immunohistochemically deficient for MSH2 and MSH6 with high levels of MSI [6], and another reported the Muir–Torre variant of Lynch syndrome, where individuals have defects in either *MSH2* or *MLH1* [6,7]. In the first case, there was no mention of tumor morphology [6], whereas in the second, the neoplasm was described as an adenocarcinoma with focal serous papillary features [7]. In the second case, there was no mention of testing the tumor for MSI or conducting immunohistochemistry for MMR proteins. A third report described a case of gastric-type adenocarcinoma of the cervix in a patient with Lynch syndrome secondary to a germline mutation in the *MSH6* MMR gene. However, MSI analysis was not performed [8].

To the best of our knowledge, this is the first case of Lynch syndrome-related clear cell carcinoma of the cervix. Clear cell carcinomas of the cervix are rare and have occurred in two distinct situations: in association with in utero diethylstilbestrol (DES) exposure [9] and sporadically [10]. Our patient had not been exposed to DES, so this case was not thought to be caused by DES exposure.

The present case emphasizes the significance of examining a patient’s cancer genetics in hereditary cancer syndromes, especially if certain cancers conflict with known facts about the syndrome. Although Lynch syndrome is most often linked to colorectal and endometrial tumors, MMR gene mutation carriers commonly develop tumors at other sites at higher rates compared to those observed in the general population [5,11,12]. Therefore, further studies should examine the contribution of MMR deficiency to tumorigenesis and include prospectively examined epidemiological data, tumor tissue, and tissue obtained following risk-reducing surgery in MMR gene mutation carriers, without discrimination against the involved organ. It is possible that cervical cancer in MMR gene mutation carriers may arise from the activation of tumorigenic pathways following human papilloma virus exposure. However, it is prudent to determine which organs are at higher risk of carcinogenesis with MMR deficiency. Such knowledge will allow for the identification of high-risk Lynch syndrome patients and the development of risk management strategies (individual and familial) and treatment regimens.

In summary, we describe the first case of Lynch syndrome-related clear cell carcinoma of the cervix, which indicates the possibility of an association between cervical cancer and Lynch syndrome. Suitable genetic tests for cancers are needed to determine if common genetics can account for synchronous or subsequent malignancies in patients with Lynch syndrome and their families. Such knowledge will enhance our understanding of the genetic mechanisms underlying apparently unrelated cancers.

## Abbreviations

MMR	mismatch repair
MLH1	MutL Homolog 1
MSH2	MutS Homolog 2
MSH6	MutS Homolog 6
PMS2	PMS1 Homolog 2
MSI	microsatellite instability
CA	carbohydrate antigen
CT	computed tomography
DES	diethylstilbesterol

## References

[1] Lynch H.T., Snyder C.L., Shaw T.G., Heinen C.D., Hitchins M.P. (2015). Milestones of Lynch syndrome: 1895–2015. Nat. Rev. Cancer.

[2] Baglietto L., Lindor N.M., Dowty J.G., White D.M., Wagner A., Gomez Garcia E.B., Vriends A.H., Cartwright N.R., Barnetson R.A., Farrington S.M. (2010). Risks of Lynch syndrome cancers for MSH6 mutation carriers. J. Natl. Cancer Inst..

[3] Senter L., Clendenning M., Sotamaa K., Hampel H., Green J., Potter J.D., Lindblom A., Lagerstedt K., Thibodeau S.N., Lindor N.M. (2008). The clinical phenotype of Lynch syndrome due to germ-line PMS2 mutations. Gastroenterology.

[4] Bonadona V., Bonaiti B., Olschwang S., Grandjouan S., Huiart L., Longy M., Guimbaud R., Buecher B., Bignon Y.J., Caron O. (2011). Cancer risks associated with germline mutations in *MLH1*, *MSH2*, and *MSH6* genes in Lynch syndrome. JAMA.

[5] Dowty J.G., Win A.K., Buchanan D.D., Lindor N.M., Macrae F.A., Clendenning M., Antill Y.C., Thibodeau S.N., Casey G., Gallinger S. (2013). Cancer risks for *MLH1* and *MSH2* mutation carriers. Hum. Mutat..

[6] Mongiat-Artus P., Miquel C., Flejou J.F., Coulet F., Verine J., Buhard O., Soliman H., Teillac P., Praz F. (2006). Spectrum of molecular alterations in colorectal, upper urinary tract, endocervical, and renal carcinomas arising in a patient with hereditary non-polyposis colorectal cancer. Virchows Arch..

[7] Nair N., Curtin J.P., Mittal K., Hiotis K.L. (2012). Cervical adenocarcinoma in a patient with Lynch syndrome, Muir-Torre variant. J. Clin. Oncol..

[8] Moat M., O’Donnell R.L., McCluggage W.G., Ralte A., Edmondson R.J. (2014). Gastric-type adenocarcinoma of the cervix in a patient with Lynch syndrome: A case report. Gynecol. Oncol. Rep..

[9] Herbst A.L. (2000). Behavior of estrogen-associated female genital tract cancer and its relation to neoplasia following intrauterine exposure to diethylstilbestrol (DES). Gynecol. Oncol..

[10] Kaminski P.F., Maier R.C. (1983). Clear cell adenocarcinoma of the cervix unrelated to diethylstilbestrol exposure. Obstet. Gynecol..

[11] Ryan S., Jenkins M.A., Win A.K. (2014). Risk of prostate cancer in Lynch syndrome: A systematic review and meta-analysis. Cancer Epidemiol. Biomark. Prev..

[12] Win A.K., Lindor N., Jenkins M. (2013). Risk of breast cancer in Lynch syndrome: A systematic review. Breast Cancer Res..

